# Genetic evidence of enzootic leishmaniasis in a stray canine and Texas mouse from sites in west and central Texas

**DOI:** 10.1590/0074-02760160225

**Published:** 2016-10

**Authors:** Evan J Kipp, Jacqueline Mariscal, Rodrigo X Armijos, Margaret Weigel, Kenneth Waldrup

**Affiliations:** 1 University of Texas at El Paso, Department of Public Health Sciences, Human Immunology & Nutrition Research Laboratory, El Paso, Texas; 2 Texas Department of State Health Services, Zoonosis Control, Health Service Regions 9/10, El Paso, Texas; 3 Indiana University, School of Public Health, Department of Environmental Health, Bloomington, Indiana

**Keywords:** Leishmania mexicana, enzootic, canines, rodent, Texas

## Abstract

We detected *Leishmania mexicana* in skin biopsies taken from a stray canine (*Canis familiaris*) and Texas mouse (*Peromyscus attwateri*) at two ecologically disparate sites in west and central Texas using polymerase chain reaction (PCR). A single PCR-positive dog was identified from a sample of 96 stray canines and was collected in a peri-urban area in El Paso County, Texas. The PCR-positive *P. attwateri* was trapped at a wildlife reserve in Mason County, Texas, from a convenience sample of 20 sylvatic mammals of different species. To our knowledge, this represents the first description of *L. mexicana* in west Texas and extends the known geographic range of the parasite to an area that includes the arid Chihuahuan Desert. Our finding of *L. mexicana* in *P. attwateri* represents a new host record and is the first description of the parasite in a wild peromyscid rodent in the United States.

Human cutaneous leishmaniasis, caused by protozoal parasites of the genus *Leishmania*, is a serious public health problem and the cause of significant morbidity throughout the world, with a preponderance of the disease burden occurring in the world’s tropical and subtropical nations (Alvar et al. 2012, Reithinger et al. 2007). Although infrequently reported, sporadic cases of autochthonous human cutaneous leishmaniasis caused by *L. mexicana* have been described in south and central Texas throughout much of the twentieth century, with an apparent increase in incidence seen in recent years (Clarke et al. 2013). In 2008, a cluster of nine autochthonous human cases was reported in the northern portion of the state in areas surrounding the urban cities of Dallas-Fort Worth (Wright et al. 2008). Recent case reports and predicted vector and reservoir distribution modeling studies have suggested that transmission of *L. mexicana* across a larger and more northern geographic range in the United States is possible in coming years due to a variety of anthropogenic and ecological factors (*i.e.*, climate change, urbanisation, and human encroachment) and having the potential to further increase incidence of the disease (McHugh et al. 1996, González et al. 2010, Clarke et al. 2013). In order to mitigate the potential for new human cases, it is essential that future studies attempt to investigate enzootic transmission of *L. mexicana* in established and emerging foci across these regions.

To date, all autochthonous cases of human cutaneous leishmaniasis in Texas have been linked to rodent reservoirs of the genus *Neotoma* and the phlebotomine sand fly vectors *Lutzomyia anthophora* and *Lu. diabolica* (McHugh et al. 1993, 1996, 2003, Clarke et al. 2013). *L. mexicana* has been detected in wild populations of *N. micropus* and *N. floridana* across south-central and east Texas and has been found in *N. albigula* in Pima County, Arizona (Kerr et al. 1999, McHugh et al. 2003). The existence of other enzootic foci in the intervening area between southern Arizona and central Texas (an ecologically diverse region that includes the arid Chihuahuan Desert and the urban city of El Paso, Texas) has not yet been investigated. In addition to *L. mexicana*, zoonotic visceral leishmaniasis caused by *L. infantum* has also been reported in the United States in domestic foxhound populations (Petersen & Barr 2009). Although transplacental transmission of *L. infantum* among canines has been predominantly reported, competent *Lutzomyia* vector species in parts of the southern United States have the potential to establish vector-borne transmission of *L. infantum* to other mammalian species, including humans, in future years (Petersen & Barr 2009, Boggiatto et al. 2011).

We attempted to find evidence of enzootic transmission of *Leishmania spp.* in two ecologically disparate sites in west and west-central Texas by searching for evidence of infection in mammalian species not currently regarded as epidemiologically relevant reservoirs. We chose to screen a group of stray canines collected in El Paso County, Texas, for the presence of either *L. mexicana* or *L. infantum.* A small sample sylvatic mammals was also made available for study and was screened for *L. mexicana*. Here, we describe our results.

In this study, we used polymerase chain reaction (PCR) to test skin biopsies from a group of stray canines (*Canis familiaris*) from El Paso County, Texas (n = 96), and a group of sylvatic mammals from Mason County, Texas (n = 20), for the presence of *Leishmania spp*. Between October 2011 and July 2012, 96 stray dogs were made available for the study following euthanasia by the City of El Paso Animal Services for routine animal control purposes (in accordance with state and local regulations). Only stray dogs collected by City of El Paso Animal Services and with no identifiable owner were included in the study. Due to logistical and legal barriers, dogs surrendered by their owners for euthanasia were excluded. Data on each dog’s sex, approximate age, breed, and the Global Positioning System (GPS) location of its collection were recorded.

A group of 20 sylvatic mammals collected within the Mason Mountains Wildlife Management Area in Mason County, Texas, was also tested for the presence of *Leishmania spp*. This convenience sample included six raccoons (*Procyon lotor)*, four white-ankled mice (*Peromyscus pectoralis)*, three hispid cotton rats (*Sigmodon hispidus)*, three white-footed mice (*P. leucopus)*, two Texas mice (*P. attwateri)*, one Piñon mouse (*P. truei*), and one white-throated wood rat (*N. leucodon*) all collected in a single trap night (October 27, 2011) using Sherman and Tomahawk live traps baited with seeds. Euthanasia of trapped rodents was done with halothane gas by public health veterinarians at the Texas Department of State Health Services (Texas Parks and Wildlife Department Collection Permit: SPR-0316-066).

All stray dogs and sylvatic mammals were visually examined for the presence of ulcerative or non-ulcerative skin lesions prior to biopsy collection. Priority was given to animals with any visible skin lesions. In these cases, biopsies were intentionally collected from around the afflicted site to include lesional tissue. In animals where no lesion was observed, a biopsy of intact skin was obtained from the base of the neck near the first thoracic vertebra and between the scapulae, as intact scapular skin is known to serve as an appropriate target for leishmanial diagnosis in canids and other mammals (Madeira et al. 2009).

Skin biopsies were preserved in a DMSO/EDTA/salt solution (20% DMSO, 250 mM EDTA, NaCl saturated, pH 8.0) and stored at room temperature. Isolation of genomic DNA from preserved biopsies occurred at a later date and was done using a phenol:chloroform:iosamyl alcohol extraction protocol (Sambrook & Russell 2001). For all biopsies, the quality of extracted genomic DNA was assessed through the PCR amplification of the mammalian interphotoreceptor retinoid-binding protein gene using previously described methods and visualised with agarose gel electrophoresis (Ferreira et al. 2010).

We then attempted to amplify the *Leishmania-*specific ribosomal non-coding region, ITS1, using the primers LITSR (5’ - CTGGATCATTTTCCGATG - 3’) and L5.8S (5’ - TGATACCACTTATCGCACTT - 3’) with a 25 μL total reaction volume and run using thermocycler conditions previously described (El Tai et al. 2000). After visualising ITS1 PCR products on ethidium bromide-stained agarose gels, we were able to identify one PCR-positive skin biopsy from each sample. Amplicons were then bidirectionally sequenced using the same ITS1 primer pair. Sequences were analysed using Basic Local Search Alignment Tool (BLAST^®^) (http://blast.ncbi.nlm.nih.gov/Blast.cgi) and a multiple sequence alignment was created using Clustal W. ITS1 sequences from *L. mexicana* (Strain M379) and *L. major* (Friedlin Strain) were included in the alignment as controls. Analysis of ITS1 sequences for both PCR-positive samples indicated significant sequence homology with *L. mexicana* reference strain M379 (Figure).

**Figure:**
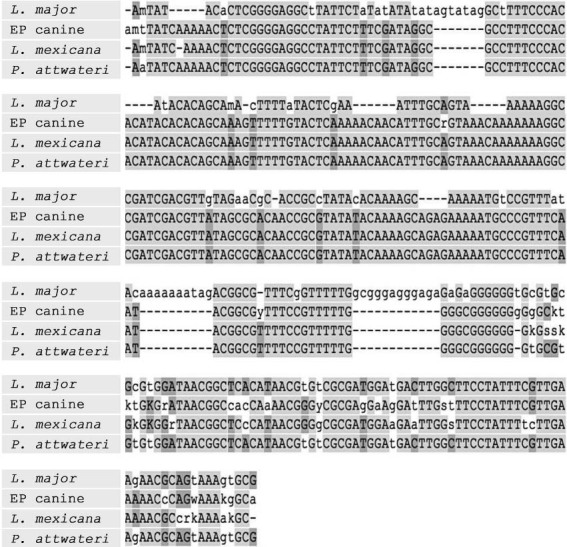
Multiple sequence alignment of ITS1 sequence from El Paso Country stray canine (EP canine) and Mason Country Texas mouse (*Peromyscus attwateri*). Included as controls are ITS1 sequences from *Leishmania mexicana* (M379) and *L. major* (Friedlin) reference strains. Areas with greatest sequence identity are shaded.

Among the 96 stray canines from El Paso County that were tested, we were able to confirm the presence of *L. mexicana* in one dog for an overall PCR prevalence of 1%. The infected dog was an adult female pit-bull terrier that was collected in a predominantly peri-urban and agricultural area adjacent to the city of El Paso (Collection GPS coordinates: 31.666480 X -106.302836). It was noted that this animal presented with a visible cutaneous lesion on its dorsum and tissue from this lesion was included in the skin biopsy. Among the convenience sample of 20 sylvatic mammals from Mason County, Texas, we were able to identify one with evidence of *L. mexicana* infection, an adult male Texas mouse (*P. attwateri)*. This particular mouse showed no evidence of any cutaneous lesion and *L. mexicana* was detected from a biopsy of intact scapular skin.

Collectively, these results suggest that enzootic transmission of *L. mexicana* is occurring across a large portion of the state of Texas and in a variety of ecological settings. We identify two new potential foci of infection in far west Texas in the arid scrubland of the Chihuahuan Desert and in central Texas in the wooded savannah of the Edwards Plateau. Our findings represent the first description of *L. mexicana* in west Texas and extend the geographic range of the parasite into the arid Chihuahuan Desert and nearby to El Paso, Texas, an urban city with approximately 650,000 inhabitants. The infected stray canine we discovered likely represents an animal that was incidentally infected with *L. mexicana.* The study area is known be inhabited by reservoir species *N. micropus* and *N. albigula*, among other rodentia, which may serve as primary reservoirs. Although no studies have been conducted on phlebotomine diversity in the region, the area is likely to be inhabited by either *Lu. anthophora* or *Lu. diabolica* and which are likely responsible for vector-borne transmission of the parasite (González et al. 2010). Our finding of *L. mexicana* in *P. attwateri* from Mason County, Texas, represents a new host record for the parasite. Although the small sample size and variety of sylvatic species contained in the sample inhibits the drawing of any meaningful epidemiologic conclusion, this finding does suggest a potentially significant new host for the parasite. Future studies are needed to better address the role of *P. attwateri* and other non-neotomid rodent species in enzootic transmission of *L. mexicana* throughout the southern United States. More epidemiologic studies are also needed to continue to identify new potential foci of this emerging zoonosis and to better understand the dynamics of its transmission in order to mitigate the potential for human infection.
